# Inferring duplications, losses, transfers and incomplete lineage sorting with nonbinary species trees

**DOI:** 10.1093/bioinformatics/bts386

**Published:** 2012-09-03

**Authors:** Maureen Stolzer, Han Lai, Minli Xu, Deepa Sathaye, Benjamin Vernot, Dannie Durand

**Affiliations:** ^1^Department of Biological Sciences; ^2^Lane Center for Computational Biology; ^3^Department of Computer Science, Carnegie Mellon University, Pittsburgh, PA 15213, USA; ^4^Department of Genome Sciences, University of Washington, Seattle, WA 98195, USA

## Abstract

**Motivation:** Gene duplication (D), transfer (T), loss (L) and incomplete lineage sorting (I) are crucial to the evolution of gene families and the emergence of novel functions. The history of these events can be inferred via comparison of gene and species trees, a process called reconciliation, yet current reconciliation algorithms model only a subset of these evolutionary processes.

**Results:** We present an algorithm to reconcile a binary gene tree with a nonbinary species tree under a DTLI parsimony criterion. This is the first reconciliation algorithm to capture all four evolutionary processes driving tree incongruence and the first to reconcile non-binary species trees with a transfer model. Our algorithm infers all optimal solutions and reports complete, temporally feasible event histories, giving the gene and species lineages in which each event occurred. It is fixed-parameter tractable, with polytime complexity when the maximum species outdegree is fixed. Application of our algorithms to prokaryotic and eukaryotic data show that use of an incomplete event model has substantial impact on the events inferred and resulting biological conclusions.

**Availability:** Our algorithms have been implemented in Notung, a freely available phylogenetic reconciliation software package, available at http://www.cs.cmu.edu/~durand/Notung.

**Contact:** mstolzer@andrew.cmu.edu

## 1 INTRODUCTION

The phylogeny of a gene family evolving by vertical descent will agree with the associated species tree. Gene duplication, gene loss, horizontal gene transfer (HGT) or incomplete lineage sorting (ILS) can result in a gene tree that differs from the species tree (Maddison, 1997). The history of such events can be inferred through topological comparison of gene and species trees, a process called ‘reconciliation’. Reconciliation encompasses two related problems: event inference and tree inference. Given rooted gene and species trees, a mapping from extant genes to extant species, and an event model, the goal of ‘event inference’ is to infer the association between ancestral genes and species and the optimal event history with respect to a combinatorial or probabilistic optimization criterion. A complete solution must include the specific events and the gene and species lineages in which those events occurred. Given a set of gene trees, ‘tree inference’ seeks the species tree that optimizes the combined events resulting from reconciliation with each gene tree in the input set.

Here, we address the event inference problem for a model that captures all four evolutionary processes contributing to gene tree incongruence. Whole genome sequencing data are revealing an ever growing number of cases where all four processes are active (e.g., Andersson, 2009; Serres *et al.*, 2009; Zhaxybayeva and Doolittle, 2011), leading to calls for algorithms that model multiple evolutionary processes (Degnan and Rosenberg, 2009; Edwards, 2009). Algorithms lacking a model of incongruence due to ILS will overestimate the number of duplications and/or transfers. For example, a recent analysis, based on a model that did not consider ILS, reported an inexplicable but dramatic increase in duplications in recently sequenced mammalian genomes (Milinkovitch *et al.*, 2010). For large-scale analysis of multigenome phylogenetic datasets, reconciliation algorithms that allow ILS to be distinguished from other sources of incongruence are essential.

### 1.1 Related work

Gene tree incongruence has been considered from two perspectives. Multispecies coalescent models focus on ILS as a source of incongruence (reviewed in Degnan and Rosenberg, 2009). The basic assumption underlying this work is that gene tree incongruence arises from ILS due to genetic drift, although some methods also take hybridization and/or recombination into account (reviewed in Degnan and Rosenberg 2009; Edwards 2009). The multispecies coalescent explicitly relates the probability of an incongruent gene tree to the time between species divergences and the effective size of the ancestral population. In the context of tree inference, these parameters can be inferred from a collection of gene trees. Event inference, however, requires prior estimates of population parameters because only one tree is under consideration.

In contrast, reconciliation focuses on incongruence that arises from processes that change the number of loci in a gene family; i.e. duplication, loss and transfer. Most event inference algorithms consider either gene duplication or HGT (Doyon *et al.*, 2011; Nakhleh, 2010; Nakhleh *et al.*, 2009), but not both. Exact algorithms with exponential time complexity have been presented for the duplication-transfer (DT) (Tofigh *et al.*, 2011) and duplication-transfer-loss (DTL) models (David and Alm, 2011), under a parsimony criterion. Event inference with transfers is NP-complete (Hallett *et al.*, 2004), but can be solved in polynomial time under a restricted model where only transfers between contemporaneous species are considered. This model (reviewed in Doyon *et al.*, 2011; Huson and Scornavacca, 2011) requires estimates of speciation times, which are frequently not known. In addition, algorithms for this restricted model may fail to recognize transfers if they involve a taxon missing from the dataset (Huson and Scornavacca, 2011; Nakhleh, 2010).

Reconciliation implicitly assumes that inter-speciation times are sufficiently long that genetic drift and incomplete lineage sorting may be safely excluded from consideration. This assumption breaks down when the species tree contains polytomies or very short branches. In these situations, allelic variation can survive multiple speciation events, leading to gene trees with branching patterns that differ from the species tree. Such cases are increasingly common due to increased sequencing of closely related species. Methods that do not consider ILS will incorrectly interpret incongruence arising from ILS as evidence of duplication or transfer.

To avoid this problem, algorithms that can distinguish between ILS and other events are needed. In fact, one parsimony criterion that considers ILS has been proposed: minimization of the number of extra gene lineages on a species branch due to Deep Coalescence (MDC) has been used as a criterion for tree inference (Maddison, 1997; Maddison and Knowles, 2006; Maddison and Maddison, 2011; Page, 1998; Than and Nakhleh, 2009). However, the MDC criterion assumes ‘all’ incongruence is due to ILS. MDC is not a suitable basis for event inference because it cannot distinguish between extra lineages arising from ILS and those arising from duplication or transfer (Zhang, 2011). Two approaches to the event inference problem combine ILS with gene duplication and loss in a single model (DLI). In earlier work, we presented the first event inference algorithm for the DLI model under a parsimony criterion (Vernot *et al.*, 2008). An event inference algorithm for a DLI model based on the multispecies coalescent relates the probability of ILS to branch lengths and population sizes explicitly (Rasmussen and Kellis, 2012). These models have different strengths. The model based on the coalescent captures more detail, but is limited to the small number of datasets for which estimates of ancestral population sizes and speciation times are available. To our knowledge, no reconciliation algorithms that consider ILS and transfer are in existence.

### 1.2 Our contributions

We present the first reconciliation algorithm for a DTLI event model that captures all four major causes of gene tree incongruence. Our algorithm is also the first to allow transfers in reconciliation with a non-binary species tree. Our algorithm is based on a simple, elegant model that recognizes ILS as a source of incongruence, but avoids the computational overhead of a full coalescent model and does not require estimates of ancestral population sizes and speciation times.

Our parsimony-based algorithm reconciles a binary gene tree with a non-binary species tree and distinguishes between incongruence that could only arise through duplication or HGT and incongruence that can be more parsimoniously explained by ILS. Our algorithm places no restriction on speciation times and reports all optimal reconciliations that are temporally feasible. For a fixed k*, the time complexity of our algorithm is *O*(*h*_S_|*V*_G_||*V*_S_|^2^) time, where k* is the out-degree of the largest polytomy in the species tree, *h*_S_ is the height of the species tree and |*V*_G_| and |*V*_S_| are the number of vertices in the gene and species trees, respectively. Given a binary species tree, our algorithm infers histories under the DTL model.

Both the DTL and DTLI algorithms have been implemented in Java and integrated in Notung, a freely available software package for phylogenetic reconciliation. Our software offers a unique and comprehensive combination of functions: it includes losses in the optimization criterion, does not require estimates of speciation times and reports all optimal event histories. Reported solutions are complete, temporally feasible event histories, giving the gene and species lineages in which each event occurred.

To demonstrate the advantages of a full-DTLI model on real data, we applied our algorithm to two phylogenetic datasets that have been used in previous analyses of HGT and phylogenetic incongruence (Delsuc *et al.*, 2005; Rokas *et al.*, 2003; Zhaxybayeva *et al.*, 2009). First, if no incongruent trees have patterns that could be most parsimoniously explained as ILS, then models with and without ILS should give same results. In fact, we observed just the opposite. The models that did not correct for ILS substantially overestimated duplications and transfers. A recent study using a quartet decomposition approach reported several highways of gene transfer between specific pairs of cyanobacterial species (Bansal *et al.*, 2011). We observed the same highways using the DTL algorithm. Only one of these highways remained when using the DTLI algorithm. Second, because many published algorithms do not include losses in the optimization criterion (e.g., Berglund *et al.*, 2006; Ma *et al.*, 2000; Tofigh *et al.*, 2011; Zmasek and Eddy, 2001), we compared models with losses (DTLI, DTL) and without losses (DTI, DT). Explicit inclusion of losses in the optimization function resulted in substantial changes to the inferred ratio of duplications to transfers, suggesting that the practice of *post hoc* inference of losses should be revisited.

Finally, when the event model includes transfers, the minimum cost event history is not, in general, unique. All algorithms cited above report only one of possibly many optimal solutions. We applied our algorithm to assess the extent to which multiple optimal solutions occur. We discovered that multiple optimal solutions are a frequent occurrence, especially in datasets where transfer is the dominant process. In the analysis reported here, 20% of 1128 cyanobacterial trees had multiple optimal solutions with inconsistent event histories. In other words, for one in five trees, the arbitrary selection of a single optimal solution could lead to conclusions that might not be supported by other optimal solutions. The results presented here are exciting and important, as they demonstrate that degeneracy and the applied event model have substantial impact on the histories inferred and, hence, on the resulting biological conclusions.

### 1.3 Notation

Given a tree, *T_i_* = (*V_i_*, *E_i_*), *L*(*T_i_*) designates the leaf set of *T_i_*, and *ρ_i_* designates its root. We use *g* ∈ *V*_G_ and *s* ∈ *V*_S_ to represent genes and species, respectively. *T_i_*(*v*) is the subtree of *T_i_* rooted at *v* ∈ *V_i_*. *C*(*v*) and *P*(*v*) denote the children and the parent of *v*, respectively, with *c_j_* ∈ *C*(*v*) denoting the *j*th child of *v*. We adopt the notation that if (*u*, *v*) ∈ *E_i_*, *P*(*v*) = *u*. Given nodes *u*, *v* ∈ *V_i_*, if *u* is on the path from *v* to *ρ*, then *u* is an ancestor of *v*, designated *u* ≥*_i_ v*, and *v* is a descendant of *u*, designated *v* ≤*_i_ u*. If *v* ≱*_i_ u* and *u* ≱*_i_ v*, *u* and *v* are ‘incomparable’, designated *u*≸*_i_v*.

## 2 ALGORITHMS

Here, we propose a reconciliation model based on DTL parsimony that distinguishes between regions of the species tree where ILS is likely, and those where only gene duplication and transfer need be considered. These differences are specified using a non-binary species tree: at binary nodes, we assume that ILS is so rare that incongruence is always evidence of gene duplication or transfer. At polytomies, ILS is considered, and gene duplication and transfer are invoked only if topological disagreement cannot be explained by ILS. This model can be invoked for both non-binary species trees and for binary species trees with short branches where ILS is suspected: even when the binary branching order of the species tree is known, the user can collapse edges in the species tree to indicate in which lineages ILS should be considered as an alternate hypothesis.

A key aspect of our model is that even when ILS is allowed, it is not possible to explain all incongruence in terms of ILS, even in a uniquely labeled gene tree. Let *g* be a node in *T_G_* and let *s* ∈ *V*_S_ be the associated node in the species tree. We wish to determine whether the divergence at *g* is consistent with a co-divergence at *s* or whether it can only be explained by events that give rise to a new locus; i.e. duplication and transfer. If the branch point at *g* arose through a co-divergence with *s*, then each species lineage descending from *s* should inherit at most one descendant of *g*. The presence of more than one descendant of *g* indicates that the divergence at *g* must be due to acquisition of an additional locus by duplication or transfer. An operational test for detecting more than one descendant on a branch results from the observation that any branching pattern that is consistent with a binary resolution of the polytomy can be explained by lineage sorting.

**Fig. 1.**
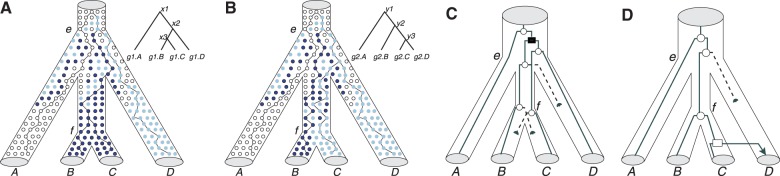
Reconciliation of binary gene trees with a non-binary species tree under our DTLI model. **(A)** A binary gene tree that is consistent with a binary resolution of the species tree. The divergences at *x*_1_ and *x*_2_ are consistent with ILS. **(B)** A gene tree that does not correspond to any binary resolution of the species tree. Node *y*_2_ is not consistent with deep coalescence: the embedding requires two descendants of *y*_2_ on the branch from *e* to *f*, a violation of model constraints. This can only be explained by persistent polymorphism (light and dark dots) on a long branch. DTLI reconciliation of the gene tree in **(B)** with the non-binary *T*_S_ results in two optimal solutions for suitable choices of *δ*, *λ* and *τ* : **(C)** one duplication followed by three losses and **(D)** one transfer and a loss. Duplications are represented by a Filled boxes, speciations by open circles, transfers by open boxes and arrows, and losses by dashed lines and filled half-circles. Each dot represents an allele of a single individual, with the dot's color indicting the type of allele. Rows represent generations of individuals

For example, the gene tree in Figure 1a represents a valid, binary resolution of the species tree, consistent with ILS. The embedding of the gene tree in the species tree shows that each species tree lineage inherits exactly one descendant of *x*_1_ and at most one descendant of *x*_2_. Both *x*_1_ and *x*_2_ can be interpreted as deep coalescences. In contrast, there is no binary resolution of the species tree that corresponds to the gene tree in Figure 1b. The embedding of this gene tree requires two descendants of *y*_2_ in the lineage from *e* to *f*, a violation of model constraints. The only way to explain two descendants of *y*_2_ on the branch from *e* to *f* is by inferring a duplication (Fig. 1c) or a transfer (Fig. 1d).

Before introducing our algorithm, we discuss the meaning of a polytomy in our model. A species polytomy can be considered from two perspectives: a ‘hard’ polytomy represents simultaneous divergence of three or more populations. A ‘soft’ polytomy represents a binary branching process in which the branching order is unknown. Our model assumes that a polytomy represents rapid or simultaneous species divergence. However, it also admits a useful interpretation for soft polytomies. A soft polytomy can be viewed as a set of hypotheses, namely the set of binary resolutions of the polytomy. Our model offers a conservative stance: events are only inferred when the topology of the gene tree does not correspond to any of these hypotheses. Note that in some cases, the hard and soft polytomy models are closely linked: the branching order of species that arose through multiple speciations in rapid successions (Ebersberger *et al.*, 2007; Pollard *et al.*, 2006) is often difficult to resolve.

### 2.1 The DTLI algorithm

In our DTLI model, divergence in a gene tree arises through one of four events: duplication (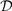), transfer (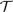), speciation (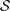) and deep coalescence (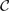). The score of a reconciliation under this model is the weighted sum of the number of duplications (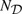), losses (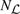), and transfers (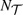):
(1)
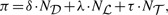

where *δ*, *λ* and *τ*, respectively, are the costs of duplication, loss and transfer. Speciation and deep coalescence represent co-divergence with binary nodes and polytomies, respectively, in the species tree and have zero cost. We refer to the cost of event *ε* ∈ {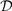, 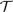, 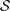, 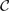} as *κ* (*ε*).

A rooted, binary gene tree *T*_G_; a rooted, arbitrary species tree *T*_S_; a mapping *M*_L_ : *L*(*V*_G_) → *L*(*V*_S_) from contemporary genes to the species from which they were sampled and a set of permitted events are given as input. The reconciliation of *T_G_* with *T_S_* results in an annotated tree, *R*_GS_ = (*V*_G_, *E*_G_), in which every internal node, *g*, is annotated with the species *s* ∈ *V*_S_ that contained gene *g*, designated *M*(*g*), and the event that caused the divergence at *g*, designated 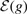. In addition, every *g* ∈ *V*_G_ \ {*ρ*_G_} is annotated with 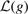, the genes lost on the edge from *P*(*g*) to *g*. Each loss is labeled with the species in which the loss occurred. We say (*u*, *v*) ∈ *E_G_* is a transfer edge if 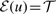 and *M* (*u*)≸_S_ *M*(*v*) and define Λ(*R*_GS_) ⊂ *E*_G_ to be the set of transfer edges in *R*_GS_. If (*u*, *v*) ∈ Λ(*R*_GS_), a transfer occurred from donor species *d* = *M*(*u*) to recipient species *r* = *M*(*v*).

Here, we present the DTLI event inference problem under the constraint that a deep coalescent is inferred at *g* if each lineage descending from *M*(*g*) inherits at most one descendant of *g*:

**The DTLI event inference problem**

**Input:** A rooted non-binary species tree, *T*_S_; a rooted, binary gene tree, *T*_G_; the leaf mapping, *M*_L_.
**Output:** All reconciliation histories *R*_GS_ that minimize *π* and satisfy the model constraints.

Algorithms for the DTLI event model must address several issues that do not arise when only a subset of the events is considered: (1) there may be more than one combination of duplications, transfers and losses that gives rise to the same pattern of tree incongruence (i.e. there may be more than one optimal solution, *R*_GS_). (2) The value of *M*(*g*) is not uniquely determined by the children of *g* and multiple possible values of *M*(*g*) must be considered because transfers cause genes to jump to distant locations in the species tree. (3) An optimal reconciliation at the root may entail a suboptimal reconciliation at an internal node, *g*. Inferring a more costly event at *g* may change the values of *M*(·) in nodes ancestral to *g* such that the overall score is reduced. Therefore, the values of *M*(*g*) and 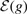 required for an optimal solution cannot be determined using only local information, and more than one optimal solution may result.

To accommodate these requirements, it is necessary to enumerate all possible assignments of *M*(*g*) and 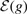, for each node *g* ∈ *V*_G_. At each *g*, the associated information is stored in two tables, 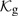 and 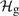. For each candidate assignment *s* ∈ *V*_S_, the score that minimizes the cost of reconciling *T*_G_(*g*) with *T*_S_(*s*), is stored in 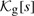. The associated events and other information needed to reconstruct the history at *g* are stored in 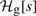.

Optimal reconciliations are calculated by a two-pass algorithm. The first pass (Algorithm 2.1.1) is a dynamic program that populates each 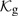 and 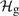 in a post-order traversal of *T_G_*. It returns the optimal reconciliation score, the values of *M*(*ρ_g_*) and 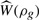 corresponding to that score and the number of optimal histories. The second pass (Supplementary Algorithm S1.0.1) is a traceback algorithm that reads information from each 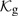 to construct an optimal solution. Each optimal history is generated by traversing, in pre-order of *T_G_*, each unique path that leads to the optimal label(s) in 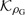. Appropriate values of *M*(*g*) and 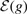 at each node *g* are selected from 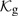. Each candidate optimal history is then tested for temporal feasibility, as described in the next section. Only those histories that are temporally feasible are reported.

A key calculation in the dynamic program of firstPass is determination of the possible events at *g* for a given candidate species assignment, *M*(*g*)= *s*. These events, in turn, depend on *M*(*c*_1_) = *s*_1_ and *M*(*c*_2_) = *s*_2_, where *c*_1_, *c*_2_ ∈ *C*(*g*). The basis for determining candidate events that are consistent with *s*, *s*_1_ and *s*_2_ is the following observation: if a duplication occurred at *g*, then the species that inherit the descendants of *c*_1_ and the species that inherit the descendants of *c*_2_ will not be disjoint.

We define a test, based on this observation, for distinguishing duplication from other events:
(2)
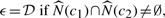

where 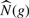 is the set of species that vertically inherit descendants of *P*(*g*). If 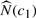 and 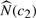 are disjoint, than one of the other three events (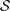,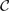 or 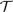) must have occurred. These events can be distinguished from one another using 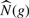, *M*(*g*) and *M*(*c*_1_) and *M*(*c*_2_), as seen in costCalc in Algorithm 2.1.1. Note that Equation (2) is different from the standard least common ancestor (lca) test; however, when *M*(*g*)= *s* is binary, the descendants of *s* are partitioned into two sets, the left and right descendants of *s*, if there is no duplication. Therefore, Equation 2 is equivalent to lca reconciliation (Vernot *et al.*, 2008).

Because 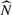 only consists of elements that were vertically inherited, we must exclude transfer edges in the calculation. For this purpose, we define

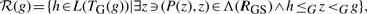

the set of leaves of *T*_G_(*g*) that were acquired through HGT. Formally, we define 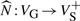 to be a mapping from *V*_G_ to sets of nodes in *V*_S_, where *V*_S_^+^ is the powerset of *V*_S_. 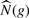 is the set of children of *M*(*P*(*g*)) such that 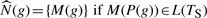; otherwise, 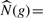

(3)
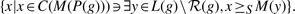


One more piece of machinery is needed: to determine 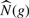, we must know the children of *M*(*P*(*g*)), but we do not have that information until we visit *P*(*g*). Therefore, we define a similar set mapping, 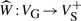, to aid in the calculation of 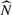. 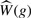 is the, set of children of *M*(*g*) that vertically inherit a descendant of *g*. Formally, if *M*(*g*) ∈ *L*(*T_S_*), 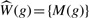; otherwise, 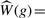

(4)
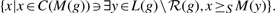


Algorithm 2.1.1 traverses *T*_G_ in post-order calling calcCost at each *g* ∈ *V_G_*. The challenge in the DTLI model is to determine the sets of species that inherit the descendants of *c*_1_ and *c*_2_ when *M*(*g*) = *s* is a polytomy; i.e. how to calculate 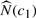 and 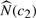. When *s* is binary, the descendants of *s* are easily partitioned into two sets; when *s* is a polytomy, all possible ways to partition the descendants must be considered. Each child of *g* can be retained in any subset of the children of *s*, ranging from size 1 to |*C*(*s*)| − 1. Our DTLI algorithm addresses this by considering all ways of partitioning *C*(*s*) into two non-empty subsets.

At each internal node *g*, the algorithm assesses all possible values for *M*(*g*) and 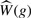 by looping through all (*s*_1_, *s*_2_) ∈ *V*_S_ × *V*_S_ and all 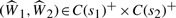. Considering all power sets corresponds to considering all the ways to partition *C*(*s*_1_) and *C*(*s*_2_). The optimal event and child mapping under *s* and 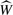 is determined by minimizing the cost of the candidate solution at *g*:
(5)
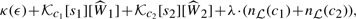

where 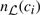, the number of losses on edge (*g*, *c_i_*), is calculated using the loss heuristic in (Vernot *et al.*, 2008). Note that for each *s*, the local cost and history tables are also indexed by all possible values of 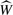, which are in *C*(*s*)^+^.

### 2.2 Temporal infeasibility

Because the donor and recipient species of any transfer must have coexisted, each transfer implies a temporal constraint. A reconciliation is temporally feasible if an ordering of species exists that satisfies the constraints of all inferred transfers. Because reconciliations inferred by Algorithm 2.1.1 are not guaranteed to be feasible, each candidate optimal solution is tested for feasibility *post hoc*.

To determine whether a reconciliation *R*_GS_ is temporally feasible, we construct a directed timing graph *G_t_* = (*V*_t_, *E*_t_) that encodes all temporal constraints on species in *T*_S_. Only species that are the donor, *d*, or recipient, *r*, of a transfer edge in Λ(*R*_GS_) must be considered. Thus, the vertex set is defined as *V_t_* = {*v* ∈ *V*_S_| ∃(*g*, *h*) ∈ Λ(*T*_G_) ∋ *v* = *M*(*g*) ∨ *v* = *M*(*h*)}.

The edges in *E_t_* represent three types of temporal constraints:

1. If species *s_i_* is an ancestor of species *s_j_* in *T*_S_, then *s_i_* predates *s_j_*: for every (*s_i_*, *s_j_*) in *V_t_* × *V_t_*, add (*s_i_*, *s_j_*) to *E_t_* if *s_i_* ≥_S_ *s_j_*.
2. Let (*g, h*) and (*g*′, *h*′) be transfers in Λ(*R*_GS_), such that *g* ≥_G_ *g*′. Then *d* = *M*(*g*) and *r* = *M*(*h*) must have occurred no later than both *d*′ = *M*(*g*′) and *r*′ = *M*(*h*′). We add (*P*(*d*), *d*′), (*P*(*d*), *r*′), (*P*(*r*), *d*′) and (*P*(*r*), *r*′) to *E_t_* .
3. Given a transfer (*g, h*) ∈ Λ(*R*_GS_), species *M*(*g*) and *M*(*h*) must be contemporaneous. Furthermore, any species that predates *M*(*g*) must also predate *M*(*h*) and vice versa. For every (*s_i_*, *s_j_*) ∈ *V_t_* × *V_t_*, add (*s_i_*, *s_j_*) to *E_t_* if ∃*s_k_* ∈ *V_t_* such that *s_i_* ≥_S_ *s_k_* and *s_k_* and *s_j_* are the donor and recipient, or vice versa, of some transfer (*g, h*) ∈ Λ(*R*_GS_).

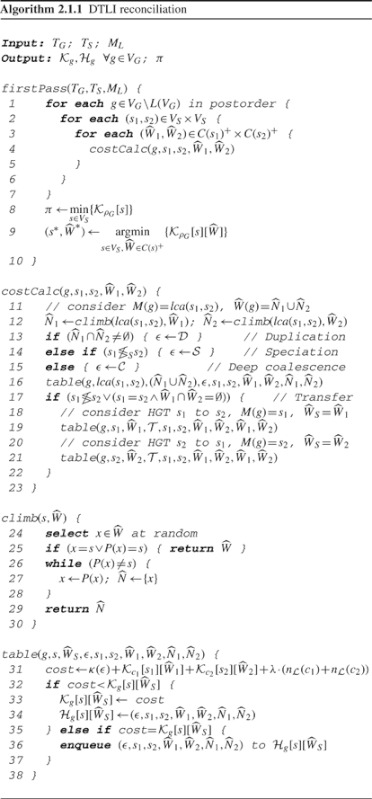


We test each candidate optimal history for temporal feasibility by verifying that the associated timing graph *G_t_* is acyclic, using a modified topological sorting algorithm in Θ (|*V_t_* |+|*E_t_* |) (Cormen *et al.*, 1990). Temporally infeasible histories are not reported. Note that it is not the case that if one optimal history is infeasible, all optimal histories are infeasible. Finding the optimal, temporally feasible reconciliation is NP-complete (Tofigh *et al.*, 2011); we leave the problem of obtaining an optimal, feasible solution when all candidate solutions have infeasible timing constraints for future work.

### 2.3 Complexity and running time

Our algorithm is fixed-parameter tractable with polynomial complexity when the size of the largest polytomy, k*, is fixed. In practical data analyses, k* is likely to be small. Recent genome-scale analyses of ILS have focused on species trees with k* = 3 (Ebersberger *et al.*, 2007; Pollard *et al.*, 2006). In general, event inference will not yield informative results when the species tree is highly unresolved.

Theorem 2.1. *Given a binary gene tree T_G_ and a non-binary species tree T_S_, firstPass takes O*(|*V_G_*|(|*V_S_*|+ n_k_ 2^k*^)*^2^*(*h_S_* + k*)) *time*.

Proof. firstPass visits each *g* ∈ *V_G_* in post order. At each *g*, costCalc is called once for every (*s*_1_, *s*_2_) ∈ *V_S_* × *V_S_* and 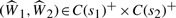, resulting in a total of *O*(|*V_G_*|(|∪*_s∈V_s__ C*(*s*)^+^|)^2^) calls to costCalc. Because |*C*(*s*)^+^| = 2^|*C*(*s*)|^ is *O*(1) when *s* is binary, |∪*_s∈V_s__ C*(*s*)^+^| is bounded above by |*V_S_*|– *n_k_* + *n_k_*2^k^*^^ and the number of calls to costCalc is *O*(|*V_G_*|(|*V_S_*|+ *n_k_*2^k^*^^)^2^). We precalculate lca(*s*_1_, *s*_2_) and test whether *s*_1_≸*s*_2_, for all species pairs, in *O*(|*V*_S_|^2^) time. Therefore, the complexity of costCalc is dominated by the calculations of 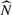 for *l* and *r*, 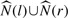 and 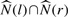. These values can be computed in *O*(*h_S_*), *O*(log(k*)) and *O*(k*) time, respectively. Thus, each call to costCalc has complexity *O*(*h_S_* + k*). Once the post-order traversal is completed, we extract the minimum score in 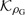, and all values of *M*(*ρ*_G_) and 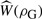 corresponding to that score. Since 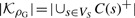, a linear search accomplishes this in *O*(|*V_S_*|+ *n_k_*2^k^*^^) time. Thus, the total complexity is *O*(|*V*_G_|(|*V*_S_|+ *n_k_*2^k^*^^)^2^(*h*_S_ + k*)).

Theorem 2.2. *secondPass returns each optimal reconciliation in O*(|*V*_G_|(*h_S_* + k*)).

Proof. secondPass starts from the *M*(*ρ*_G_) and 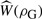 found in firstPass. It then constructs an optimal solution by visiting each subsequent *g* ∈ *V_G_*, assigning mappings and events by looking up values in 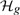 in constant time. Losses are inferred in *O*(k* + *h*_S_) time (see Vernot *et al.*, 2008). Thus, the complexity for returning each optimal history is *O*(|*V*_G_|(*h*_S_ + k*)).

When *T_S_* is binary, firstPass is completed in *O*(*h*_S_|*V*_G_||*V*_S_|^2^) time, and secondPass reports each optimal solution in *O*(*h*_S_|*V*_G_|) time.

Our Notung implementation is efficient in practice. We measured the time required to reconcile 1128 cyanobacterial gene trees with a species tree of size |*V*_S_| ≤ 21 for all the parameter settings given in Table 1. To assess the effect of polytomy size, we also collapsed edges in the species tree to create a polytomy ranging in size from 2 to 6. The maximum average running time observed on a single AMD Opteron 2.3 ghz, 64-bit processor was ~ 0.05s. per solution.

**Table 1.** Event counts for the cyanobacteria dataset, with *δ* = 3 and *λ* = 2

Model	*τ*	*n_D_*	*n_T_*	*n_L_*	*n_C_*	Infeasible	Degenerate
DT	2.5	7	1798	1560	0	84	6
DT	6	1648	191	6096	0	0	0
DT	10	2066	0	7520	0	0	0
DTI	2.5	6	1521	1468	559	3	67
DTI	6	1425	133	5133	595	0	0
DTI	10	1691	0	5921	636	0	0
DTL	2.5	0	2121	781	0	42	13
DTL	6	73	1740	1516	0	82	50
DTL	10	1324	480	4797	0	83	40
DTLI	2.5	0	1783	895	409	92	16
DTLI	6	82	1458	1456	542	90	109
DTLI	10	1122	405	4093	602	4	53

Event counts from 314 gene trees. Temporally infeasible or conflicting degenerate solutions in any model were removed. The number of trees not considered for each model and setting is given in the last two columns, respectively.

## 3 EMPRICAL RESULTS

To assess the importance of a four-event model, we implemented our DTLI algorithm in Notung2.7 and applied it to two phylogenetic datasets in which ILS, HGT and hybridization have been studied (Bansal *et al.*, 2011; Yu *et al.*, 2011). Because a number of algorithms and software packages do not include losses in the optimization criterion, we sought to assess the impact of this modeling choice. Therefore, we also implemented and applied models excluding losses in the optimization criterion (DT and DTI) models. Except where stated, the trends reported here were observed consistently in both datasets.

**Fig. 2.**
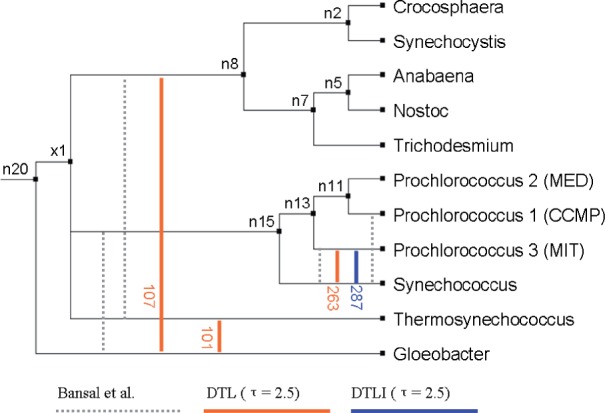
Predicted transfer highways using the DTL and DTLI models with *δ* = 3, *τ* = 2.5 and *λ* = 2. Predicted highways with transfer counts exceeding 1.5 standard deviations above the mean are shown, with the total number of transfers labeled. Highways predicted by Bansal *et al.* (2011) are shown as dashed lines

The datasets analyzed contain 1128 cyanobacterial gene trees sampled from 11 species (Figs 2 and Supplementary Fig. S1), and 106 yeast gene trees sampled from 15 species (Supplementary Fig. S2), respectively. Each gene tree has at most one gene copy per species. To assess the impact of our ILS model, for each dataset we compared the performance of our algorithm on a binary and a non-binary species tree. The non-binary species tree was created by removing one edge resulting in a single polytomy of size 3. In each case, the selected edge was short and associated with substantial gene tree incongruence. Each polytomy was chosen as a reflection of an area of the species tree where ILS may be occurring. In both cases, the selected edge was one that is reportedly difficult to resolve (Bansal *et al.*, 2011; Schirrmeister *et al.*, 2011; Yu *et al.*, 2011).

We reconciled each tree using each of the four models (DT, DTI, DTL and DTLI), with *τ* ∈ {2.5,6,10}, *δ* = 3 and *λ* = 2 (when considered). We tabulated (1) the number of events of each type, (2) the gene and (3) species lineages in which they occurred, (4) the donor and recipient of each transfer and (5) the number of temporally infeasible reconciliations (Table 1 for cyanobacteria; Supplementary Table S1 for yeast). Trees that had no temporally feasible solution for at least one set of parameter values were eliminated from analysis under all models and values of *τ*. For each setting, gene trees were rooted with Notung's rooting optimization algorithm using event parsimony. If a tree had multiple optimal solutions (one or more optimal roots or reconciliations for a specified root), it was only retained if all solutions yielded the same counts for each event.

Our observations highlight the extent to which model choice and degeneracy affect biological inferences. Approximately 10% of trees were removed because they are potentially misleading due to temporal infeasibility. Hallett *et al.* (2004) reported no temporal infeasibility for the application of their DT algorithm to a simulated dataset. Our results suggest that infeasible cases can be more prevalent in real data.

In addition, ~ 20% of trees had conflicting optimal solutions, suggesting that inferences based on a single, randomly selected optimal solution could lead to conclusions that are not, in fact, supported by the data. This result highlights the importance of taking multiple solutions into account when performing tree reconciliation.

When the models with and without ILS are compared, we observed a substantial decrease in the combined number of duplications and transfers, ranging from 15% to 18% in cyanobacteria and 11% to 14% in yeast. We also observed considerable decreases in the number of losses, as high as 20% in the case of DT versus DTI. These differences indicate the extent to which ignoring ILS can lead to overestimation of other events.

Recently, great interest has been focused on ‘highways’ of HGT (pairs of species with very active genetic exchange, relative to HGT in other species) [i.e. (Bansal *et al.*, 2011; Beiko *et al.*, 2005)]. We considered evidence of HGT highways in our cyanobacterial data, where a highway is an outlier in the total number of transfers, in both directions, between a pair of species. With the DTL model, we observe traffic (Fig. 2, red lines) similar to the HGT highways reported by Bansal *et al.* (2011) (dotted lines), for the same dataset. However, when events were inferred with the DTLI model, the elevated transfer rates in the Gloeobacter group disappeared, resulting a single highway (blue line). These results demonstrate that use of a complete event model is crucial for accurate inference.

In general, including losses in the optimization criterion resulted in (1) a dramatic decrease in the number of losses and (2) a change in the ratio of the number of duplications to transfers. This likely occurs because duplications and losses are coupled. When losses are included in the optimization, their cost may prevent the model from over-inferring duplications. This suggests that for any application where accurate reconstruction of event histories matters, including losses in the optimization criterion is crucial.

## 4 DISCUSSION

This work presents the first reconciliation algorithm for the event inference problem under a model that captures the four major evolutionary processes driving tree incongruence: duplication, loss, transfer and ILS. Our algorithm reconciles a binary gene tree with a non-binary species tree and is, to our knowledge, the first algorithm to allow non-binary species trees with a transfer model. Our algorithm outputs detailed event histories, describing the specific events inferred and the lineages in which they occurred.

When restricted to binary species trees, our algorithm reduces to an event inference algorithm for the DTL model that can infer all optimal solutions and does not require estimates of speciation times or otherwise restrict transfers to a limited set of species pairs.

Algorithms that capture duplication, transfer and ILS in a single, integrated model are of increasing importance (Degnan and Rosenberg, 2009). New sequencing technologies are leading to rapid growth of whole genome datasets, in which there is evidence for both HGT and ILS. Our empirical analyses of two different datasets, representing both prokaryotic and eukaryotic data, indicate that use of a complete event model has substantial impact on the events inferred and, hence, the resulting biological conclusions. For example, it is possible that apparent HGT highways could be, at least in part, mis-interpretations of deep coalescence.

Our model is a compromise between current reconciliation models, which ignore ILS everywhere, and coalescent models that explicitly relate the probability of incongruence to the length and population size associated with every branch. Our model is more expressive than the former and more efficient and more widely applicable than the latter. A great strength of the multispecies coalescent is that it explicitly relates the probability of incongruence to effective population size and the time between species divergences. Estimates of these population parameters are only available for a limited set of well-studied species. However, given a sufficiently large set of gene families, population parameters can be inferred directly from the data, but this is computationally demanding. For example, species tree inference from a set of 106 genes in 8 yeast species required 800 h using Bayesian estimation on a coalescent model, whereas a parsimony method inferred the identical tree in only a ‘fraction of a second’ (Than and Nakhleh, 2009).

A parsimony model, on the other hand, does not take branch lengths into account, resulting in a potential reduction in accuracy. Future simulation studies are planned to characterize the accuracy of this approach. The benefits of this simpler model are that it can be applied to any set of taxa, not just species for which population parameters can be estimated, and it is not sensitive to overfitting. Because it is fast and general, it is highly suitable for processing large, genome-scale datasets.

The work presented here could profitably be generalized in several ways, including a model of transfers in which multiple genes are transferred in a single event; inference methods for datasets involving extinct or missing species; and ILS models that deviate from the assumption of a uniform gene tree distribution and take branch lengths and population size into account for datasets where such information is available. Another important area for future work is the selection of event costs and investigation of the robustness of results with respect to small changes in the costs used. Note that the problem of how to weight events also arises in coalescent models. For example, the coalescent-based DLI inference algorithm requires the user to supply duplication and transfer rates.
